# Predictors for quality of life improvement after surgery for degenerative cervical myelopathy: a prospective multi-center study

**DOI:** 10.1186/s12955-021-01789-7

**Published:** 2021-05-19

**Authors:** Hiroyuki Inose, Takashi Hirai, Toshitaka Yoshii, Atsushi Kimura, Katsushi Takeshita, Hirokazu Inoue, Asato Maekawa, Kenji Endo, Takuya Miyamoto, Takeo Furuya, Akira Nakamura, Kanji Mori, Shunsuke Kanbara, Shiro Imagama, Shoji Seki, Shunji Matsunaga, Atsushi Okawa

**Affiliations:** 1 Department of Orthopaedic and Trauma Research, Tokyo Medical and Dental University, 1-5-45 Yushima, Bunkyo-Ku, Tokyo 113-8519 Japan; 2 Department of Orthopedic Surgery, Tokyo Medical and Dental University, Bunkyo-Ku, Japan; 3 Department of Orthopaedics, Jichi Medical University, Shimotsuke, Japan; 4 Department of Orthopaedic Surgery, Tokyo Medical University, Shinjuku-Ku, Japan; 5 Department of Orthopedic Surgery, Chiba University Graduate School of Medicine, Chiba-shi, Japan; 6 Department of Orthopaedic Surgery, Shiga University of Medical Science, Otsu, Japan; 7 Department of Orthopedic Surgery, Nagoya University Graduate School of Medicine, Nagoya, Japan; 8 Department of Orthopedic Surgery, Faculty of Medicine, University of Toyama, Toyama, Japan; 9 Department of Orthopedic Surgery, Imakiire General Hospital, Kagoshima-shi, Japan

**Keywords:** Degenerative cervical myelopathy, Quality of life, European QOL-5 Dimensions, Surgery, Spinal cord compression, Lumbar lordosis, Sacral slope, T1 pelvic angle

## Abstract

**Background:**

Degenerative cervical myelopathy (DCM) can significantly impair a patient’s quality of life (QOL). In this study, we aimed to identify predictors associated with QOL improvement after surgery for DCM.

**Methods:**

This study included 148 patients who underwent surgery for DCM. The European QOL-5 Dimension (EQ-5D) score, the Japanese Orthopedic Association for the assessment of cervical myelopathy (C-JOA) score, and the Nurick grade were used as outcome measures. Radiographic examinations were performed at enrollment. The associations of baseline variables with changes in EQ-5D scores from preoperative to 1-year postoperative assessment were investigated using a multivariable linear regression model.

**Results:**

The EQ-5D and C-JOA scores and the Nurick grade improved after surgery (*P* < 0.001, *P* < 0.001, and *P* < 0.001, respectively). Univariable analysis revealed that preoperative EQ-5D and C-JOA scores were significantly associated with increased EQ-5D scores from preoperative assessment to 1 year after surgery (*P* < 0.0001 and *P* = 0.045). Multivariable regression analysis showed that the independent preoperative predictors of change in QOL were lumbar lordosis (LL), sacral slope (SS), and T1 pelvic angle (TPA). According to the prediction model, the increased EQ-5D score from preoperatively to 1 year after surgery = 0.308 − 0.493 × EQ-5D + 0.006 × LL − 0.008 × SS + 0.004 × TPA.

**Conclusions:**

Preoperative LL, SS, and TPA significantly impacted the QOL of patients who underwent surgery for DCM. Less improvement in QOL after surgery was achieved in patients with smaller LL and TPA and larger SS values. Patients with these risk factors may therefore require additional support to experience adequate improvement in QOL.

## Introduction

Degenerative cervical myelopathy (DCM) is the most common cause of spinal dysfunction in adults [1]. DCM is caused by age-related changes in the spine, including degeneration of the facet joints, discs, and/or vertebral bodies, progressive spinal kyphosis, and ossification, calcification, or thickening of the spinal ligaments [1]. These anatomical changes narrow the spinal canal, resulting in progressive spinal cord compression, neurological deterioration, and a significant decline in quality of life (QOL). Naturally, as a patient ages, cervical spine degeneration is expected to progress and the pressure on the spinal cord from degenerative tissue will increase. Accordingly, as the population ages, the number of patients with DCM is increasing [2].

DCM is a progressive disease, although there are limited reports of clinically significant functional improvements with conservative treatment [3]. On the other hand, there are several reports of improvement in function and QOL following surgery for DCM [4–6]. However, it remains unclear which patients experience poor QOL improvement after surgery. To date, most of the reports on surgical outcomes have used the Japanese Orthopedic Association score for the assessment of cervical myelopathy (C-JOA) to evaluate neurological status [7–9]. However, based on international assessment standards, there are several problems associated with C-JOA scores including that (1) they do not reflect the patient's self-assessment of pain, numbness, and health status and (2) they are assessed primarily from the physician's perspective [10]. There is still insufficient evidence regarding the extent of improvement in QOL after surgery for DCM using validated patient-based assessments [11]. This knowledge gap makes it difficult to determine the appropriate indications for and timing of surgical treatment to manage DCM, especially in individuals with severe QOL impairments. Therefore, this study aimed to identify the factors that influence the improvement of QOL after surgery for DCM patients based on European QOL-5 Dimension (EQ-5D) score assessments.

## Methods

### Study population

This multicenter study, initiated by the Japanese Organization of the Study for Ossification of the Spinal Ligament, prospectively recruited patients with DCM who were scheduled for surgical treatment at eight participating institutions between October 2016 and December 2017. DCM includes cervical spondylotic myelopathy, ossification of the posterior longitudinal ligament, and other spinal abnormalities that cause cervical cord compression. This study was approved by the institutional review board of each hospital, and written informed consent was obtained from all individual participants. In our facility, the institutional review board approved this study on June 28, 2016, under protocol number M2016-017.

Demographic data, including age, sex, body mass index, and etiology of myelopathy, were recorded. Data on the follow-up period, type of surgery, and handgrip strength were also recorded; we collected data on serum albumin levels preoperatively. The grip strength was measured once on each arm before surgery by using an analogue hand dynamometer or a digital hand dynamometer. To exclude the effect of the dominant arm, we calculated the average of the right and left handgrip strength.

The exclusion criteria included the presence of comorbidities impairing physical function (e.g., cerebral infarction, cerebral palsy, or severe inflammatory/autoimmune rheumatic diseases), bedridden status or full dependence on a wheelchair before surgery due to severe cervical myelopathy, and difficulty in completing a questionnaire due to cognitive impairment.

Surgical indications and procedures (laminoplasty, anterior decompression and fusion, posterior decompression and fusion, or anterior and posterior decompression and fusion) were determined based on characteristics of individual patients, such as neurological status, presence of anterior compression, and spinal alignment. During this period, 175 patients were scheduled for surgery for DCM. A total of 171 patients completed the 1-year follow-up; however, as 23 patients had missing EQ-5D values during the course of the study, the analysis was performed on the remaining 148 patients.

### Radiologic findings

Cervical lordosis of the spine was defined by the Cobb angle between C2 and C7 on a lateral radiograph in the neutral position. C2–7 range of motion was measured on flexion–extension lateral radiographs. The C7 slope was calculated by measuring the angle formed by the horizontal line to the C7 vertebra and the line parallel to the superior endplate of the C7 vertebra [12]. Thoracic kyphosis was defined by the Cobb angle between the superior and inferior endplates of T1–T12 [13]. The C2–7 sagittal vertical axis (C2–7 SVA) is the sagittal distance between a plumb line dropped from the center of C2 and the posterosuperior corner of C7 [14]. Lumbar lordosis (LL) was defined as the angle between the superior endplate of L1 and the inferior endplate of L5 [15]. The sacral slope (SS) was defined as the angle formed between the line of the upper end plate of the sacrum and the horizon [16]. The SVA is the sagittal distance between the C7 plumb line and the vertical line through the posterosuperior corner of the S1 endplate on standing whole-spine lateral radiographs [17]. Pelvic tilt (PT) is the angle between the vertical reference line from the center of the femoral head and the line from the center of the femoral head to the midpoint of the sacral endplate [18]. The T1 pelvic angle (TPA) is the angle between the line from the femoral head axis to the centroid of T1 and the line from the femoral head axis to the middle of the S1 endplate [19]. The presence of any lumbosacral transitional vertebra (LSTV) was evaluated based on the Castellvi method using anteroposterior radiographs [20]. Patients with type II, III, and IV LSTVs were assessed as LSTV positive. The cervical lordosis, C7 slope, and C2–7 SVA were measured on the standing lateral cervical radiographs in the neutral position. The LL, SS, SVA, PT, and TPA were measured on lateral whole-spine radiographs in the standing position. All radiographs were taken preoperatively.

### Outcome measures

The severity of neurological symptoms, walking ability, and QOL were assessed upon enrollment and 1 year after surgery using the C-JOA score, the Nurick grade, and the Japanese three-level version of the EQ-5D score (EQ-5D-3L), respectively. The Japanese version of the EQ-5D-3L score comprises five dimensions: mobility, self-care, usual activities, pain and discomfort, and anxiety and depression. The digits for these five dimensions can be combined in a 5-digit code describing the patient’s health status. The code was then converted into a Japan-specific single index value using country-specific value sets (ranges from − 0.111 to 1, with higher scores indicating better QOL) [21]. The C-JOA score evaluates six categories of function for the assessment of DCM: motor dysfunction in the upper and lower extremities (0–4), sensory function in the upper and lower extremities (0–2), sensory function in the trunk (0–2), and bladder function (0–3). The total of these subscales ranges from a minimum score of 0 to a maximum score of 17, with lower scores indicating greater severity of neurological symptoms [22]. The Nurick classification of myelopathy (Nurick grade, range: 0–5, with lower grades indicating better walking ability) was used to assess walking ability [23]. The EQ-5D-3L questionnaire was self-completed by each patient without the assistance of the surgeon. The C-JOA score and Nurick grade were evaluated by the surgeon who performed the surgery.

### Statistical analyses

We performed the Wilcoxon signed-rank test for data with a skewed distribution to identify differences in scores before surgery and 1 year after surgery after assessing normality with the Shapiro–Wilk test. Outcomes were compared among the three surgical methods (laminoplasty, anterior decompression and fusion, and posterior decompression and fusion). After assessing for normality with the Shapiro–Wilk test, we analyzed continuous variables using the Mann–Whitney U-test followed by the Steel–Dwass multiple comparison test for continuous data with a skewed distribution.

The associations between baseline variables and changes in scores (the difference from preoperative to 1 year after surgery) for EQ-5D were investigated using multivariable linear regression models. First, predictors associated with the dependent variable at a *P* value of < 0.25 in the univariable regression analyses were carried forward to the second step of the analysis [24]. Second, the remaining predictors were included in a backward stepwise multivariable regression analysis along with the baseline equivalent (preoperative EQ-5D score) of the dependent variable. Including the baseline equivalent is a standard procedure in prediction analysis because this variable is usually the most important predictor in the regression model [25]. In the regression analysis, we calculated the 95% confidence intervals for all predictive values. The number of cases during the study period determined the sample size. To measure the dispersion of the numerical variables, we calculated the standard deviation. Data are presented as means ± standard deviation or as numbers (%). For all statistical analyses, JMP version 14 (SAS Institute, Cary, NC, USA) was used, and a *P* value of < 0.05 was considered to indicate statistical significance.

## Results

### Patient demographics

A total of 148 patients were included. The baseline characteristics of the patients are shown in Table 1.

**Table 1:** Baseline characteristics of the patients

*Characteristics*	
Preoperative follow-up period (days)	52.9 ± 46.6
Postoperative follow-up period (days)	375.7 ± 51.8
Age (years)	68.2 ± 10.3
Sex (female)	59 (40)*
BMI	24.3 ± 3.7
OPLL	60 (41)*
*Type of surgery*	
Anterior decompression and fusion	32 (22)*
Laminoplasty	91 (61)*
Posterior decompression and fusion	24 (16)*
Anterior and posterior surgery	1 (0)*
Albumin (g/dL)	4.1 ± 0.5
Grip strength average	19.5 ± 8.2
*Radiographic assessments*	
C2–7 angle	12.3 ± 11.7
C2–7 ROM	28.9 ± 17.2
C2–7 SVA	23.3 ± 14.7
C7 slope	26.0 ± 11.6
TK	36.3 ± 12.9
PT	18.7 ± 7.7
TPA	18.8 ± 12.3
LL	37.3 ± 12.7
SVA	33.3 ± 42.6
SS	29.8 ± 8.1
LSTV	16 (11) *

*BMI* body mass index, *OPLL* ossification of the posterior longitudinal ligament, *ROM* range of motion, *SVA* sagittal vertical axis, *TK* thoracic kyphosis, *PT* pelvic tilt, *TPA* T1 pelvic angle, *LL* lumbar lordosis, *SS* sacral slope, *LSTV* lumbosacral transitional vertebra

*Data are expressed as numbers (%); all other values are expressed as means ± standard deviation

### Outcome measures and radiographic assessments

Table 2 shows the differences in outcome measures between preoperative assessments and 1-year postoperative assessments. EQ-5D and C-JOA scores as well as Nurick grades were significantly improved postoperatively (Table 2). We then examined whether the amount of change in EQ-5D score was different among the three surgeries (anterior decompression and fusion, laminoplasty, and posterior decompression and fusion). The results showed no significant differences in EQ-5D score and change in EQ-5D score before and after surgery among the three surgeries (Table 3).

**Table 2:** Outcome measures

*Outcome measures*		
EQ-5D		*P*
Before surgery	0.57 ± 0.18	< 0.001
1 year after surgery	0.67 ± 0.17	
C-JOA sore		
Before surgery	11.0 ± 2.6	< 0.001
1 year after surgery	13.1 ± 2.3	
Nurick grade		
Before surgery	2.7 ± 1.0	< 0.001
1 year after surgery	2.1 ± 1.2	

The Wilcoxon signed-rank test was used

*JOA* Japanese Orthopaedic Association, *EQ-5D* European Quality of Life-5 Dimensions

**Table 3:** Comparison of EQ-5D scores among groups

	Anterior decompression and fusion (N = 32)	Laminoplasty (N = 91)	Posterior decompression and fusion (N = 24)	*P* value (*P*_A–L_, *P*_A–P_, *P*_L–P_)
Preoperative EQ-5D score	0.58 ± 0.20	0.56 ± 0.14	0.60 ± 0.27	0.64, 0.99, 0.58
Postoperative EQ-5D score	0.69 ± 0.18	0.66 ± 0.17	0.70 ± 0.20	0.85, > 0.99, 0.86
Change in EQ-5D score from preoperatively to 1 year after surgery	0.11 ± 0.17	0.09 ± 0.20	0.10 ± 0.26	> 0.99, 0.90, 0.94

*P*_A–L_, *P*_A–P_, and *P*_L–P_ represent the comparisons between the anterior decompression and fusion (A), laminoplasty (L), and posterior decompression and fusion (P) groups, respectively, using the Steel–Dwass test. Plus–minus values are means ± standard deviation

*EQ-5D* European Quality of Life-5 Dimensions

### Independent predictors of increased EQ-5D score from preoperatively to 1 year after surgery

The associations between baseline variables and changes in EQ-5D scores (the difference from the preoperative assessment to 1 year after surgery) were investigated using a univariable regression model. There was a significant association between preoperative C-JOA and EQ-5D scores and increased EQ-5D scores from preoperative assessment to 1 year after surgery (Table 4). Although not statistically significant, PT, TPA, LL, SS, and the presence of LSTV tended to be associated with an increase in EQ-5D score from preoperative assessment to 1 year after surgery (*P* = 0.14, 0.07, 0.14, 0.18, and 0.09, respectively) (Table 4).

**Table 4:** Univariate regression analysis: association of baseline variables with increased EQ-5D score from preoperative assessment to 1 year after surgery

Characteristic	β	95% CI	*P*
Age (years)	− 0.001	− 0.005 to 0.002	0.38
Sex (female)	0.011	− 0.022 to 0.045	0.52
BMI	− 0.004	− 0.013 to 0.004	0.32
OPLL	0.002	− 0.031 to 0.036	0.89
Albumin (g/dL)	− 0.003	− 0.064 to 0.057	0.92
Grip strength average	0.001	− 0.003 to 0.005	0.55
EQ-5D	− 0.556	− 0.706 to − 0.406	< 0.0001
C-JOA sore	− 0.013	− 0.026 to − 0.0003	0.045
Nurick grade	0.019	− 0.015 to 0.052	0.27
C2–7 angle	− 0.001	− 0.004 to 0.002	0.48
C2–7 ROM	0.0002	− 0.002 to 0.002	0.83
C2–7 SVA	0.001	− 0.001 to 0.003	0.31
C7 slope	− 0.0005	− 0.003 to 0.002	0.74
TK	− 0.0005	− 0.003 to 0.002	0.73
PT	0.004	− 0.001 to 0.009	0.14
TPA	0.003	− 0.0002 to 0.006	0.07
LL	0.004	− 0.001 to 0.009	0.14
SVA	0.00007	− 0.0007 to 0.0009	0.86
SS	− 0.003	− 0.007 to 0.001	0.18
LSTV	− 0.04	− 0.098 to 0.008	0.09

*β* regression coefficient, *BMI* body mass index, *OPLL* ossification of the posterior longitudinal ligament, *EQ-5D* European Quality of Life-5 Dimensions, *JOA* Japanese Orthopaedic Association, *ROM* range of motion, *SVA* sagittal vertical axis, *TK* thoracic kyphosis, *PT* pelvic tilt, *TPA* T1 pelvic angle, *LL* lumbar lordosis, *SS* sacral slope, *LSTV* lumbosacral transitional vertebra

An increase in EQ-5D score from preoperative assessment to 1 year after surgery was defined as EQ-5D score at 1 year after surgery—preoperative EQ-5D score

Then the independent predictors for increased EQ-5D score from preoperative to 1 year after surgery were evaluated using a multivariable regression analysis. Based on the univariable analysis, the dependent variable was defined as the increase in the EQ-5D score from preoperative assessment to 1 year after surgery, and the candidate independent variables were preoperative EQ-5D score, PT, TPA, LL, SS, and the presence of LSTV. As a result, the independent baseline predictors were identified as LL (β = 0.006, *P* = 0.001), SS (β = − 0.008, *P* = 0.003), and TPA (β = 0.004, *P* = 0.01) (Table 5).

**Table 5:** Multiple regression analysis: independent predictors of increased EQ-5D score from preoperative assessment to 1 year after surgery

Factor	β	95% CI	*P*
EQ-5D	− 0.493	− 0.707 to − 0.279	< 0.001
LL	0.006	0.003 to 0.010	0.001
SS	− 0.008	− 0.013 to − 0.003	0.004
TPA	0.004	0.001 to 0.007	0.02

An increase in EQ-5D score from preoperative assessment to 1 year after surgery was defined as EQ-5D score at 1 year after surgery—preoperative EQ-5D score

*β* regression coefficient, *EQ-5D* European Quality of Life-5 Dimensions, *LL* lumbar lordosis, *SS* sacral slope, *TPA* T1 pelvic angle, *CI* confidence interval

**Fig. 1:**
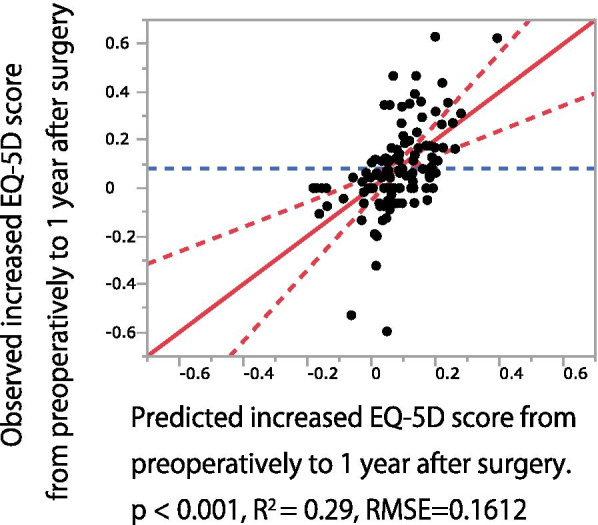
Observed versus predicted multivariable linear regression plots for increase between preoperative and 1-year-postoperative EQ-5D scores

According to this prediction model, the following equation was obtained: increase in EQ-5D score from preoperative assessment to 1 year after surgery = 0.308 − 0.493 × EQ-5D + 0.006 × LL − 0.008 × SS + 0.004 × TPA; R^2^ = 0.29 (Fig. 1). The variables in the final model were controlled for multicollinearity according to the value of each variable's variance inflation factor. These results indicate that patients with a greater LL and TPA were more likely to improve their QOL than those with a lower LL and TPA. This prediction model also revealed that patients with a small SS are more likely to have improved QOL after surgery than those with a steep SS.

## Discussion

This study investigated the predictors of QOL improvement following surgery for DCM. The EQ-5D and C-JOA scores and Nurick grade improved after surgery. Univariable analysis indicated that preoperative EQ-5D and C-JOA scores were significantly associated with an increase in EQ-5D scores from preoperative assessment to 1 year after surgery. Multivariable regression analysis revealed that the independent preoperative predictors were LL, SS, and TPA. According to the prediction model, an increase in EQ-5D score from preoperative assessment to 1 year after surgery = 0.308 − 0.493 × EQ-5D + 0.006 × LL − 0.008 × SS + 0.004 × TPA. To the best of our knowledge, this study is the first to investigate the predictive value of preoperative patient-reported outcome measures and radiographic assessments for predicting changes in QOL 1 year after surgery for DCM.

In this study, we found that the EQ-5D and C-JOA scores as well as the Nurick grade improved after surgery for DCM. Thus, surgery for DCM improved patients' QOL, neurological symptoms, and walking ability. To date, there is limited evidence that conservative treatment for cervical myelopathy is associated with improved QOL and neurological symptoms [3]. A recent systematic review revealed that without surgical intervention, 20–60% of cervical spondylotic myelopathy patients will deteriorate neurologically over time [26]. Therefore, in patients with progressive decline in QOL and neurological symptoms due to DCM, surgery should be performed. However, future prospective randomized studies need to be undertaken to confirm the superiority of surgical treatment for DCM over conservative treatment because some prospective randomized studies have been conducted for lumbar spinal stenosis [27, 28].

Univariable analysis showed that preoperative EQ-5D and C-JOA scores were significantly associated with increased EQ-5D scores from preoperative assessment to 1 year after surgery. The results of the present study are consistent with those of previous study, which showed that preoperative severity was an important predictor of surgical outcome [29]. In this study, TPA and the presence of LSTV tended to be associated with changes in QOL in the univariable regression analysis (*P* = 0.07 and 0.09). From an anatomical point of view, the presence of LSTV is known to affect SS and sacral tilt [30, 31]. In the data set of this study, the patients with LSTV had significantly higher SVA and TPA compared to the patients without LSTV, although there was no significant difference for SS (data not shown). Collectively, these results suggest an association between LSTV and global spinal sagittal alignment. The relationship between LSTV and QOL in DCM patients therefore needs further investigation.

Interestingly, multivariable regression analysis showed that LL, SS, and TPA, but not cervical spinal parameters, were independent predictors of postoperative QOL improvement. Thus, the degree of improvement in QOL after the resolution of cervical spinal cord compression in DCM patients may depend more on the thoracolumbar spinal alignment than on the cervical spine. In addition, the results of the multivariable regression analysis showed that large LL and TPA had a positive impact on postoperative QOL improvement in DCM, while large SS had a negative impact. To date, the significance of SS and TPA in the QOL of DCM patients remains unclear. SS correlates with LL [32]; in cases where the sagittal spinal balance is impaired, SS decreases to compensate for the imbalance [33]. In contrast, in cases with high SS, LL tends to be larger to balance the sagittal spinal alignment. TPA accounts for both global malalignment and compensation through pelvic retroversion, and TPA > 20 is an indicator of spinal kyphosis [19]. Therefore, the final prediction model might be interpreted as overall spinopelvic alignment including SS, LL, and TPA is more important than SS, LL, or TPA alone to achieve adequate postoperative QOL improvement in DCM patients. If future randomized controlled trials are designed to precisely compare the outcomes of cervical spine surgery, the alignment of the thoracolumbar spine as well as the cervical spine may be adjusted.

In addition, the results of this study showed that the preoperative EQ-5D value had a negative impact on the improvement of QOL after surgery. This may be attributed to the fact that in cases where the preoperative EQ-5D score is high, the postoperative improvement is calculated to be small. Indeed, a study on lumbar disc herniation that included preoperative EQ-5D values in multivariate regression analysis reported that an increase in preoperative EQ-5D scores by one unit resulted in one less postoperative EQ-5D change [34]. In view of these findings, patients with these risk factors should be advised to consider measures such as long-term postoperative rehabilitation and home modifications, as their QOL may not improve sufficiently after surgery.

Limitations of this study include the following: First, although we prospectively collected data, it is difficult to exclude bias due to the characteristics of this study cohort. Therefore, the results might not be reproducible in another cohort with different characteristics. Second, not all patients were treated with the same surgical procedure (laminoplasty, anterior decompression and fusion, posterior decompression and fusion, or anterior and posterior decompression and fusion). The stability associated with these procedures may affect QOL recovery after surgery. However, an increase in EQ-5D scores between these procedures showed no statistical difference. Therefore, in this study, we included all procedures for analysis. Third, the exclusion criteria may include factors that might potentially contribute to poor QOL improvement. Accordingly, patients with the poorest QOL improvement may have been excluded from the study. Lastly, the final prediction model shows R^2^ = 0.29. This means that this model explains 29% of EQ-5D score improvement. Therefore, various factors other than those found in this study might be involved in the improvement of QOL after surgery. Of note, regarding the interpretation of R^2^ values, Cohen et al. proposed the following threshold to interpret the magnitude of the effect sizes for R^2^ of the model (small: 0.02, medium: 0.13, and large: 0.26) [35]. According to this threshold, the effect size of the final prediction model in this study can be interpreted as large.

## Conclusions

Preoperative LL, SS, and TPA contributed significantly to improvement in the QOL of patients who underwent surgery for DCM. Additionally, the smaller the LL and TPA and the steeper the SS, the less improvement in QOL was observed. Patients with these risk factors may therefore need additional support to achieve sufficient improvement in QOL. These findings may assist physicians in selecting an appropriate treatment strategy to prevent inadequate improvement in QOL after surgery for DCM. 

## Data Availability

Not applicable.

## Abbreviations

C-JOA: Japanese Orthopedic Association for the assessment of cervical myelopathy
DCM: Degenerative cervical myelopathy
EQ-5D: European QOL-5 Dimension
LL: Lumbar lordosis
PT: Pelvic tilt
QOL: Quality of life
SS: Sacral slope
SVA: Sagittal vertical axis
TPA: T1 pelvic angle
